# Differential Regulation of Mitochondrial Pyruvate Carrier Genes Modulates Respiratory Capacity and Stress Tolerance in Yeast

**DOI:** 10.1371/journal.pone.0079405

**Published:** 2013-11-14

**Authors:** Alba Timón-Gómez, Markus Proft, Amparo Pascual-Ahuir

**Affiliations:** 1 Department of Biotechnology, Instituto de Biología Molecular y Celular de Plantas, Universidad Politécnica de Valencia, Valencia, Spain; 2 Department of Mechanisms of Plant Stress Responses, Instituto de Biología Molecular y Celular de Plantas, Consejo Superior de Investigaciones Científicas, Valencia, Spain; University of Hong Kong, Hong Kong

## Abstract

Mpc proteins are highly conserved from yeast to humans and are necessary for the uptake of pyruvate at the inner mitochondrial membrane, which is used for leucine and valine biosynthesis and as a fuel for respiration. Our analysis of the yeast *MPC* gene family suggests that amino acid biosynthesis, respiration rate and oxidative stress tolerance are regulated by changes in the Mpc protein composition of the mitochondria. Mpc2 and Mpc3 are highly similar but functionally different: Mpc2 is most abundant under fermentative non stress conditions and important for amino acid biosynthesis, while Mpc3 is the most abundant family member upon salt stress or when high respiration rates are required. Accordingly, expression of the *MPC3* gene is highly activated upon NaCl stress or during the transition from fermentation to respiration, both types of regulation depend on the Hog1 MAP kinase. Overexpression experiments show that gain of Mpc2 function leads to a severe respiration defect and ROS accumulation, while Mpc3 stimulates respiration and enhances tolerance to oxidative stress. Our results identify the regulated mitochondrial pyruvate uptake as an important determinant of respiration rate and stress resistance.

## Introduction

Mitochondria have many essential functions in eukaryotic cells. The generation of ATP by oxidative phosphorylation is their primary function, however, mitochondria play important roles in signal transduction, conversion of metabolites and the biosynthesis of organic compounds. Additionally, mitochondria are the main intracellular source of reactive oxygen species (ROS), which are produced by electron leakage from their electron transport chains [1]. As a consequence, mitochondrial ROS production is a major determinant of the chronological lifespan of the cell [2], [3]. Therefore the mitochondrial energy metabolism must be highly regulated to ensure optimal ATP supply and balanced ROS production under changing nutritional or stress conditions.

The budding yeast, *Saccharomyces cerevisiae*, serves as an important model system to understand mitochondrial regulation. In the presence of fermentable sugars, yeast cells predominantly generate ATP by glycolysis and repress mitochondrial respiration. When glucose becomes limiting for example during the diauxic shift, a major transcriptional program is initiated to activate the catabolism of non fermentable carbon sources and oxidative metabolism at the mitochondria [4]. This adaptive process is regulated by conserved signal transduction such as positively by the Snf1 signaling pathway or negatively by protein kinase A [5], [6]. As a result, yeast cells growing on non fermentable substrates show a largely enhanced mitochondrial biomass and respiratory capacity.

Mitochondrial up-regulation is not restricted to the switch from fermentation to respiration as it also occurs under other stress conditions such as osmotic stress. Mitochondrial functions have been shown to be essential for the efficient adaptation to high salinity. Under these conditions mitochondrial components are selectively activated to balance the ROS levels during stress [7], [8]. The high osmolarity glycerol (HOG) MAP kinase pathway, which specifically responds to hyperosmotic stress [9], and the Snf1 kinase have been implicated in the transcriptional up-regulation of mitochondrial functions upon osmostress [8]. Reinforcement of mitochondrial oxidative metabolism might be required because of mitochondrial damage caused by the salt stress. Interestingly, the transcriptional activators Rtg1 and Rtg3 of the retrograde signaling pathway responding to mitochondrial dysfunction have been identified as direct effectors of the HOG pathway [10].

The import of pyruvate into mitochondria is a crucial step for both biosynthesis of organic compounds and for oxidative energy metabolism. In yeast, mitochondrial pyruvate is a precursor for the synthesis of branched-chain amino acids. Alternatively pyruvate is converted into acetyl-coenzyme A (acetyl-CoA) by the pyruvate dehydrogenase complex in the mitochondrial matrix and is the major donator of carbon atoms into the citric acid cycle. Very recently the molecular nature of the mitochondrial pyruvate carrier (Mpc) has been revealed [11], [12]. In yeast, the major pyruvate uptake system at the inner mitochondrial membrane under fermentative growth contains the Mpc1 and Mpc2 proteins, which have been shown to be essential for amino acid and lipoic acid biosynthesis. The Mpc1/Mpc2 pyruvate carrier has been proposed to contain Mpc1 as the minor core subunit in a complex with several Mpc2 subunits. A minor isoform, Mpc3, exists in yeast but did not seem to contribute to the import of pyruvate dedicated to the biosynthesis of organic compounds. Here we investigate the role of the different Mpc proteins in oxidative metabolism. We find that the inducible Mpc3 protein is an important determinant of respiratory capacity. In turn, the overexpression of Mpc1 or Mpc2 negatively affected respiration. Our data are in agreement with a model were the regulated composition of Mpc proteins upon stress or during the diauxic shift adjusts the rate of pyruvate oxidation during respiration.

## Materials and Methods

### Yeast Strains and Culture Conditions

*S. cerevisiae* strains used in this study were: wild type BY4741 (*MATa*; *his3*Δ*1*; *leu2*Δ*0*; *met15*Δ*0*; *ura3*Δ*0*) and the mutant alleles *mpc1::KanMX4*; *mpc2::KanMX4*; *mpc3::KanMX4; crc1::KanMX4* [*13*] *; mpc1::KanMX crc1::HIS3 (this study); mpc2::KanMX crc1::HIS3 (this study); mpc3::KanMX crc1::HIS3 (this study); mpc1::KanMX mpc2::LEU2 mpc3::HIS3 (this study)*. Yeast strains expressing chromosomally tagged TAP fusion proteins were: BY4741 (*MATa*; *his3*Δ*1*; *leu2*Δ*0*; *met15*Δ*0*; *ura3*Δ*0*) with *MPC1-TAP-His3MX*, *MPC2-TAP-His3MX*, or *MPC3-TAP-His3MX* [14]. Yeast cultures were Δgrown in yeast extract-peptone containing 2% dextrose (YPD) or 3% glycerol (YPGlyc) with or without the indicated supplementation of NaCl. Synthetic growth medium contained 0.67% yeast nitrogen base, 50 mM succinic acid pH 5.5 and 2% dextrose (SD), 2% galactose (SGal), 2% lactate (SLac) or 3% glycerol (SGlyc). According to the auxotrophies of each strain and as indicated, methionine (10 mg/l), histidine (10 mg/l), leucine (10 mg/l), uracil (25 mg/l) or valine (10 mg/l) were added.

### Plasmids

For the overexpression driven by the constitutive GPD promoter and intracellular localization with C-terminal fused dsRed, the full length *MPC1*, *MPC2* or *MPC3* genes were subcloned into yeast expression vector pAG423-GPD-ccdB-dsRed (2 micron, *HIS3*) [15]. For the inducible expression of HA epitope tagged Mpc3, the full length *MPC3* gene was cloned into yeast expression vector pAG426-GAL1-ccdB-HA (2 micron, *URA3*) [15]. Mitochondrially directed GFP was expressed from plasmid pVT100U-mtGFP (2 micron, *URA3*) [16].

### Reverse Transcriptase Assays

Total RNA was isolated by acid phenol extraction from yeast cells grown in the indicated condition (YPD, YPGlyc or YPD+NaCl). Total RNA samples were DNaseI digested and purified with the RNeasy Mini kit (Qiagen). A total of 5 µg RNA was converted into DNA using the Superscript III first strand cDNA synthesis kit (Invitrogen). The amount of DNA was quantified with the indicated gene specific primers by quantitative PCR in real time using the EvaGreen qPCR Master Mix (Biotium) on an Applied Biosystems 7500 Sequence Detection System. The *ACT1* gene expression was used as a reference. The expression level was determined in triplicate from at least two independent cDNA samples.

### Immunological Methods

Equal amounts of total protein from whole cell extracts were separated by 10% SDS-PAGE and analyzed by immunoblotting on PVDF membranes using anti-peroxidase-anti-peroxidase (anti-PAP) antibody (Sigma; 1∶10.000) and peroxidase-labelled anti-mouse antibody (Amersham Biosciences, 1∶10.000). The bands were visualized with ECL Plus (Amersham Biosciences) and quantified with the Fujifilm LAS3000 system. DB71 staining of the membranes was used as a loading control [17].

### Continuous Growth Assays

For sensitivity assays in continuous growth, fresh overnight precultures of the indicated yeast strains were diluted in triplicate in multi-well plates to the same OD. Growth was then constantly monitored under the indicated conditions in a Bioscreen C system (Thermo) for the indicated times.

### Fluorescence Microscopy

Yeast cells containing the plasmid pVT100U-mtGFP were transformed with the indicated MPC-dsRed overexpression constructs and grown in SD medium to exponential growth phase. Cells were observed on a Leica confocal microscope TCS SL with λ_Ex_ 488 nm/λ_Em_ 500–530 nm for mitochondrial GFP and with λ_Ex_ 558 nm/λ_Em_ 583 nm for dsRed.

### Measurement of Oxygen Consumption

For measurements of respiration rates, yeast cells were grown exponentially in SD medium (Mpc overexpressing yeast strains) or SGlyc medium (*mpc* deletion strains), washed with water and finally resuspended at the same OD in 40 mM NaPO_4_ pH 7.4 with 1% glucose. Oxygen consumption was then quantified in intact cells using a Mitocell S200 Respirometry System (Strathkelvin Instruments) with a Clarke type oxygen electrode. Oxygen consumption rates were determined from at least three independent yeast cultures for each strain background.

### Quantification of ROS

Yeast cells were grown to exponential phase. Culture aliquots were incubated for 30 min with 2′,7′-dichlorodihydrofluorescein diacetate (Sigma) at a final concentration of 10 µM. The cells were washed with water and resuspended in 1 ml of 50 mM Tris/HCl pH 7.5. After the addition of 10 µl of chloroform and 5 µl of 0.1% SDS, the cells were extracted by rigorous agitation. Fluorescence was quantified in the supernatant in a Victor X5 microplate fluorescence reader (Perkin Elmer) at 492 nm excitation and 525 nm emission wavelengths and normalized for the fluorescence of the same number of mock treated cells.

### Coprecipitation Experiments

Cells (100 ml) carrying the pGAL1-Mpc3-HA expression construct in the presence of the indicated chromosomally tagged *MPC* gene were grown to exponential phase in SGal medium. Mitochondria were enriched by differential centrifugation according to the protocol described in [18]. Finally mitochondria were resuspended in 250 µl buffer A (50 mM Tris/HCl pH 7.5; 15 mM EDTA; 2 mM DTT; 150 mM NaCl) in the presence of protease inhibitors (EDTA free, Roche). Mitochondria were lysed by incubation with 2% Di-Dodecyl-maltosid (DDM) at 4°C for 20 min. Immunoprecipitation was carried out with anti-HA monoclonal antibody (12CA5; Roche) coupled to Dynabeads-protein A (Invitrogen) at 4°C for 2 hours. The beads were extensively washed with buffer A, resuspended in 1x Laemmli buffer and finally analysed by anti-HA or anti-Tap immunoblot.

## Results

### Mpc3 Protein and mRNA Levels are Highly Regulated upon Salt Stress and during Diauxic Shift

**Figure 1:**
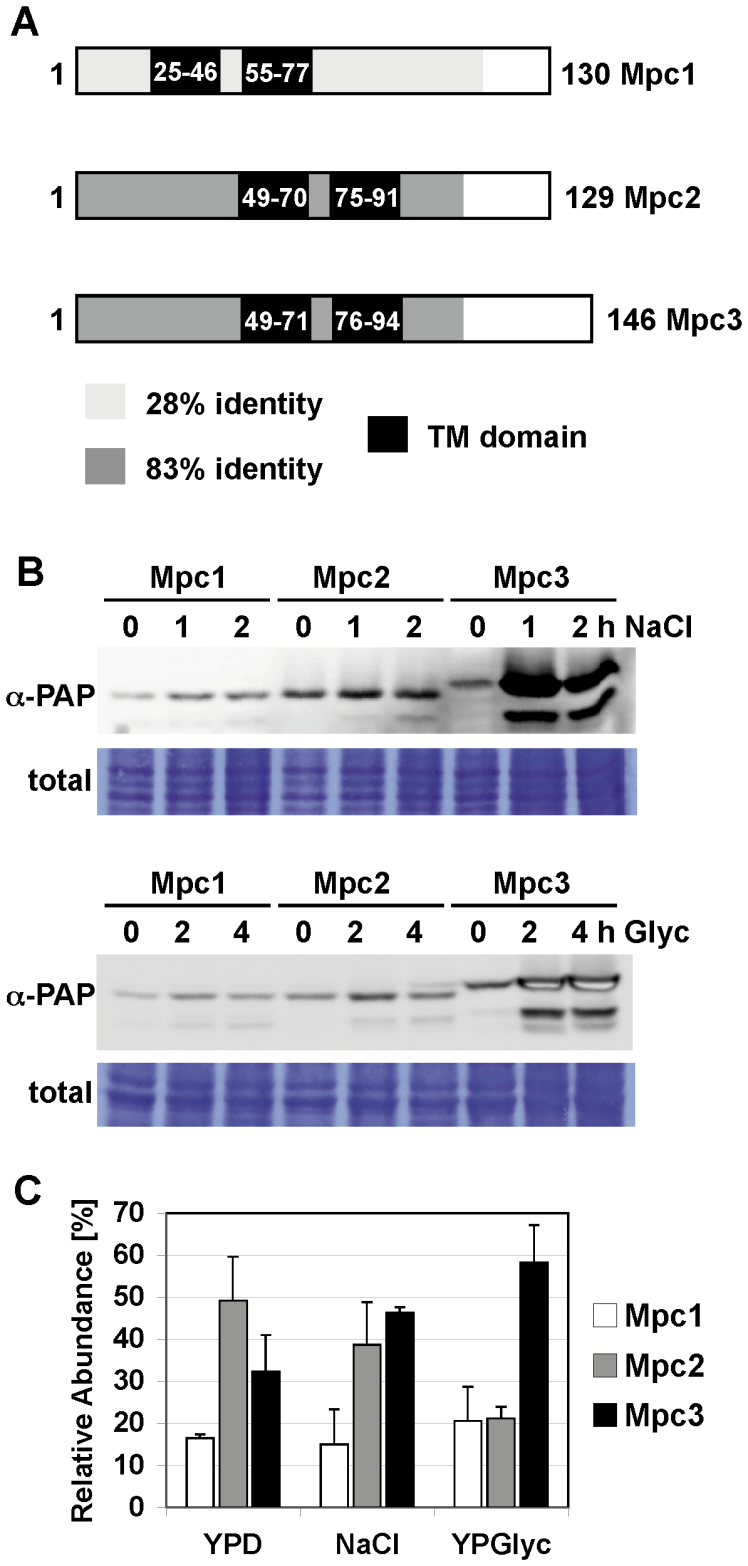
Mpc3 is a highly regulated member of the yeast *MPC* gene family. A, Amino acid sequence comparison of the yeast Mpc mitochondrial pyruvate carriers. Mpc2 and Mpc3 are 83% identical in the N-terminal 106 amino acids. Mpc1 is 28% identical to Mpc2 or Mpc3 in the N-terminal 116 amino acids. Putative transmembrane domains were identified using the TMpred tool at ch.EMBnet.org. B, Mpc3 abundance is activated by salt stress and diauxic shift. Yeast strains expressing the indicated TAP-tagged Mpc proteins from the endogenous promoters were used. Cells were subjected to osmotic stress (0.4 M NaCl) or shifted from glucose to glycerol medium for the indicated times. Mpc proteins were visualized by anti-PAP immunoblotting and compared to total protein loading. C, the relative distribution of Mpc proteins changes during adaptation to salt stress or non fermentative growth conditions. The individual Mpc protein levels were quantified as in (B) using cells continuously growing in normal medium (YPD), high salt medium (0.4 M NaCl) or glycerol medium (YPGlyc). The relative abundance of each Mpc protein was calculated under each growth condition. Data are shown as mean +/− standard deviation from three independent experiments. Mpc3 protein levels are significantly higher (p<0.05) as compared to Mpc1 or Mpc2 on glycerol medium according to the Students t-test.

The *Saccharomyces cerevisiae* genome contains three mitochondrial pyruvate carrier encoding *MPC* genes: *MPC1* (*YGL080W*), *MPC2* (*YHR162W*) and *MPC3* (*YGR243W*) [11], [12]. Mpc1 and Mpc2 have been proposed to form the major mitochondrial pyruvate uptake system for amino acid biosynthesis [11], [12]. Since loss of Mpc3 did not interfere with amino acid synthesis, its function was not further investigated. A sequence comparison of the yeast Mpc proteins reveals that Mpc2 and Mpc3 are highly homologous (Fig. 1A). Both proteins are >80% identical over most of the N-terminal region and display short C-terminal extensions which are unrelated to each other. The Mpc1 protein shares much less similarity (<30% identity) with either Mpc2 or Mpc3. Two transmembrane domains are predicted for each Mpc family member, which are likely the regions embedded in the inner mitochondrial membrane (Fig. 1A). As expected from the high degree of similarity, Mpc2 and Mpc3 show an identical location of both transmembrane helices. However, in the Mpc1 protein these domains are located closer to the N-terminus.

To gain insights into the function of the different Mpc proteins, we quantified their abundance upon different environmental conditions. Yeast strains expressing chromosomally Tap tagged versions of the Mpc proteins were used in these immunological studies. As shown in Fig. 1B, Mpc3 protein levels rapidly increased upon salt stress or during the shift from glucose to a non fermentable carbon source. The abundance of the Mpc1 and Mpc2 proteins was unaffected by the same treatments. We next determined the steady state levels of the Mpc proteins in fully adapted cells under normal, salt stress and completely respiratory conditions (Fig. 1C). We found that Mpc2 was the most abundant Mpc protein in rich glucose conditions. The relative abundance of Mpc3, however, increased upon continuous growth in the presence of high salinity. Finally, Mpc3 is the predominant family member under growth conditions which require high respiration rates. The relative abundance of Mpc1 was unaffected by the different growth conditions. Since osmotic stress causes a limited [8] and growth on glycerol a complete induction of mitochondrial respiration, these data indicated that a correlation exists between respiratory capacity and Mpc3 abundance.

**Figure 2:**
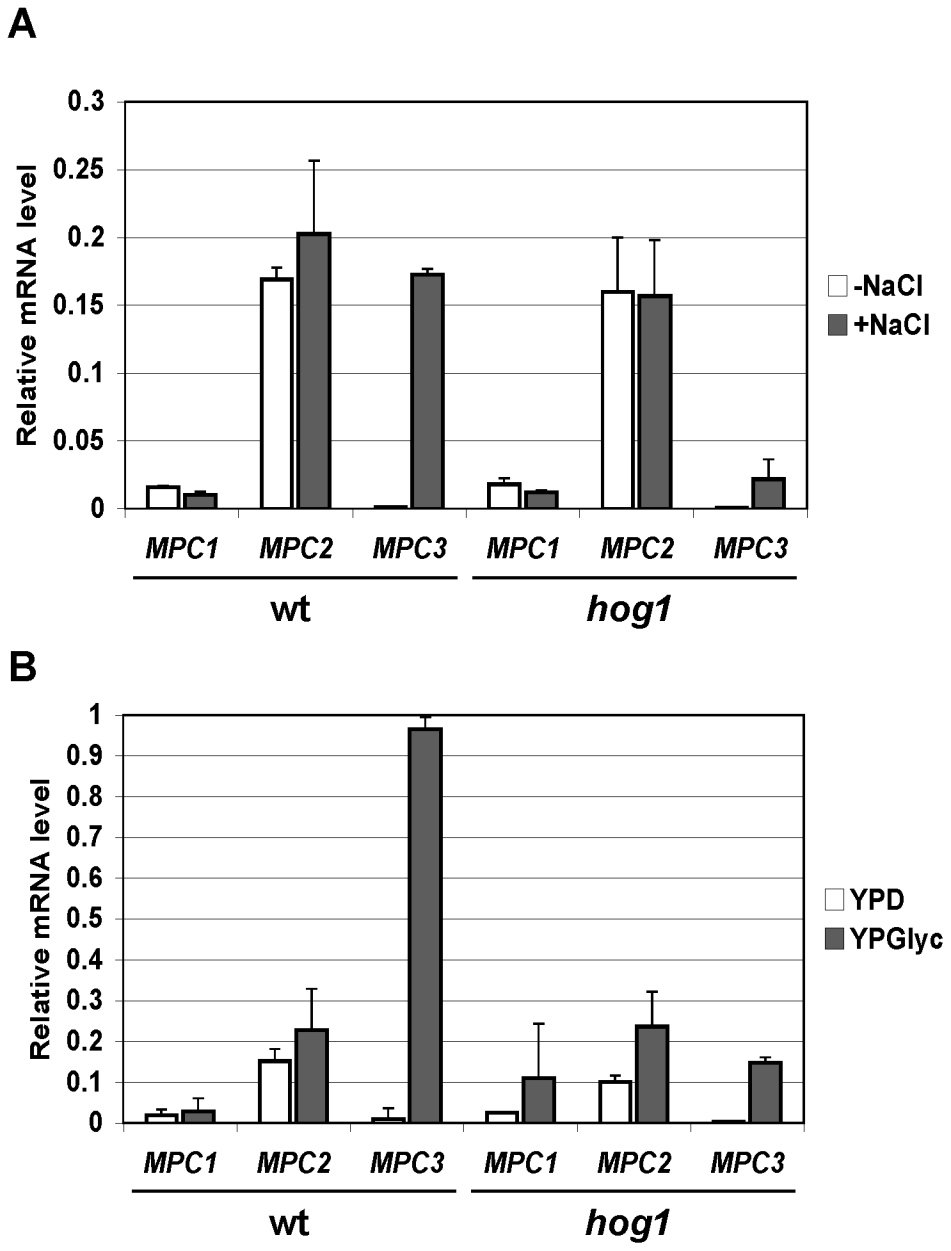
Transcriptional regulation of *MPC* genes in response to salt stress and diauxic shift. Yeast wild type (BY4741, wt) and *hog1* mutant strains were treated for 10 min with 0.4 M NaCl (A) or shifted from YPD to YPGlyc medium for 30 min (B). The expression levels were determined by RT-PCR and normalized for the *ACT1* messenger. Data are shown as mean from two independent experiments; error bars represent standard deviation. *MPC3* transcript levels are significantly lower in the *hog1* mutant vs. wild type after NaCl induction (p = 0.001) or diauxic shift (p = 0.0003) according to the Students t-test.

We next determined whether the different Mpc protein distributions could be attributed to the regulated expression of *MPC* genes. We quantified the transcriptional regulation at the three *MPC* genes by Reverse Transcriptase assays. As shown in Fig. 2, *MPC3* mRNA levels increased dramatically upon salt stress and during the shift to respiration. For *MPC1* and *MPC2* we determined constant mRNA levels independent on the stress conditions. We concluded that a strict transcriptional control of the *MPC3* gene contributes to the differential expression of Mpc proteins upon normal, osmostress and respiratory growth conditions. We finally found that the HOG pathway is needed for the efficient induction of *MPC3* transcription both upon salt stress and during the diauxic shift (Fig. 2).

### Mpc Proteins Differentially Contribute to Amino Acid Biosynthesis and Respiratory Growth

**Figure 3:**
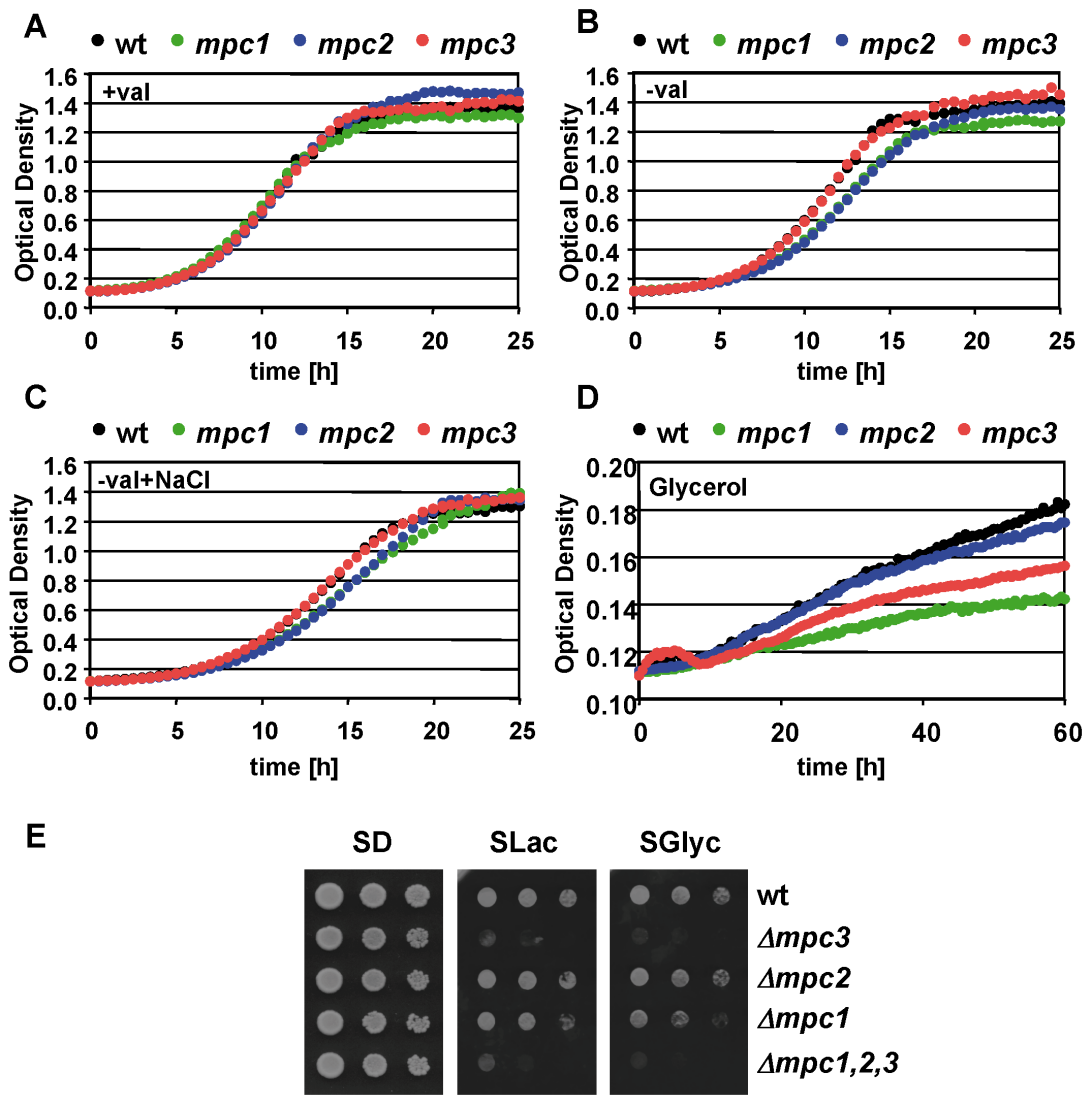
Differential requirement of Mpc proteins for amino acid biosynthesis and respiratory growth. The growth of the wild type (BY4741, wt) and the indicated mutant strains was monitored in SD medium with (A) or without valine (B), with valine in the presence of 0.4 M NaCl (C) and in SGlyc medium with valine (D). Data presented are mean values from three biological replicates. The standard deviation is 2–5% for (A), 2–4% for (B) and (C), and 0.5–1% for (D). (E) Plate assay of yeast wild type and the indicated *mpc* mutant strains upon fermentative (SD) or respiratory (SLac and SGlyc) growth conditions.

We have seen before that the relative distribution of Mpc proteins changes along with the activation of respiratory metabolism in response to environmental conditions. Mpc1 and Mpc2 are essential for the biosynthesis of leucine and valine in mitochondria [11], [12]. Therefore we investigated the contribution of different Mpc proteins to amino acid biosynthesis and respiratory growth. We compared the growth kinetics of deletion mutants for the individual *MPC* genes in medium without valine and with glycerol as the sole carbon source. Expectedly we detected a delayed growth in the absence of valine for the *mpc1* and *mpc2* mutants (Fig. 3). The loss of Mpc3 function did not cause any phenotype under the same growth conditions. Having seen that *MPC3* expression was strongly induced upon salt stress, we repeated the growth assay in the presence of NaCl to determine whether an enhanced Mpc3 level could compensate the lack of Mpc2 or Mpc1. However, high salinity did not improve the growth of *mpc1* or *mpc2* mutants without valine (Fig. 3C). These data suggested that the physiological role of Mpc3 was different from Mpc2 and not linked to amino acid biosynthesis. We further tested the importance of all three Mpc proteins for the efficient growth with a non fermentable carbon source. We found that in this case the Mpc2 function was dispensable and detected a decreased growth efficiency for the *mpc1* and *mpc3* mutants (Fig. 3D). Since the growth in synthetic glycerol medium was very slow in the microplate setup of the Bioscreen system used for the continuous growth measurements, most probably due to oxygen limitation, we additionally tested the respiratory growth of the *mpc* strains on agar plates. As shown in Figure 3E, we confirmed the clearly reduced growth rate on respiratory carbon sources lactate and glycerol for the *mpc3* and the triple *mpc1,2,3* mutants. The lack of Mpc2 function clearly also affected respiratory growth, although to a lesser extent.

### Gain of Mpc Function Differentially Affects Respiratory Capacity, Stress Resistance and ROS Production

**Figure 4:**
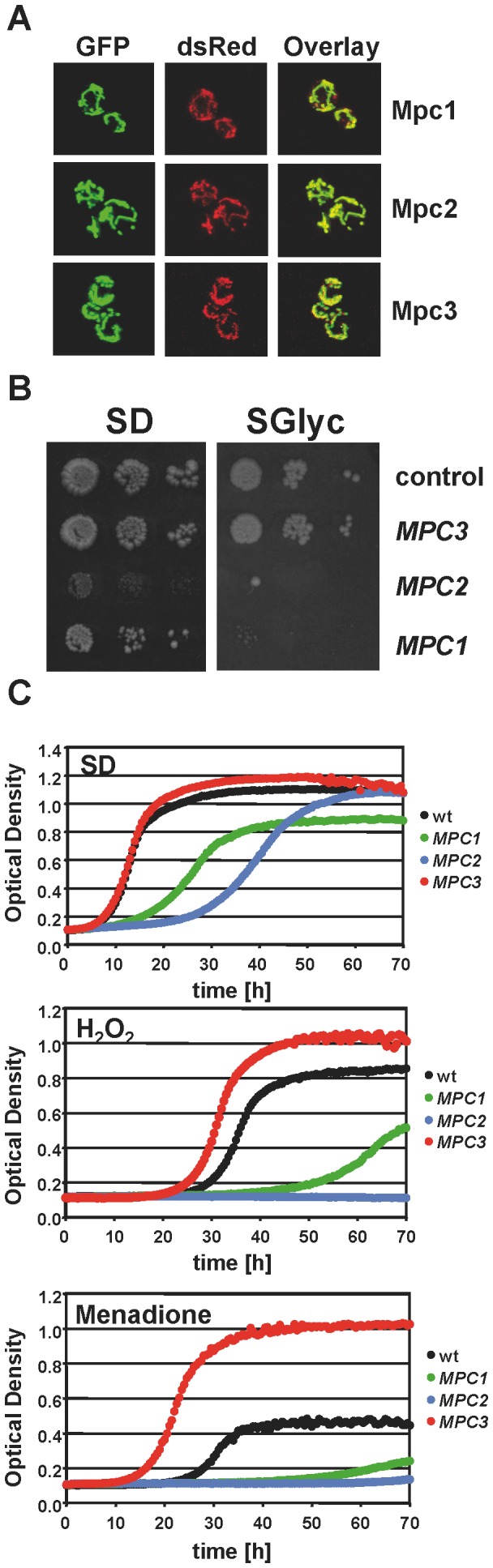
The effect of Mpc overexpression on respiratory growth and oxidative stress tolerance. Individual Mpc proteins were overexpressed as dsRed fusions in yeast wild type cells. A. All three Mpc-dsRed fusion proteins localize exclusively to the mitochondria. Yeast cells were cotransformed with the respective Mpc overexpressing constructs and the mitochondrial GFP expressing pVT100 plasmid. Representative pictures are shown for GFP, dsRed and the overlay obtained by confocal fluorescence microscopy. B. The overexpression of Mpc1 or Mpc2 inhibits respiratory growth. The growth of yeast wild type cells overexpressing the indicated Mpc proteins was assayed on glucose (SD) or glycerol (SGlyc) containing minimal medium and compared to the respective empty vector control. C. Overexpression of Mpc3 leads to oxidative stress tolerance. The growth of yeast strains described in (B) was continuously monitored in normal medium (SD) and in the presence of 2 mM hydrogen peroxide or 25 µM menadione. Data presented are mean values from three biological replicates. The standard deviation is 1–3% for SD, 1–2% for hydrogen peroxide and 2–10% for menadione growth.

As shown above, the Mpc protein composition is regulated by fermentative or respiratory growth and during salt stress. Mpc2 function appeared to be important for amino acid biosynthesis under non stress fermentative conditions, while Mpc3 function was highly inducible and important upon respiratory conditions. We next investigated the physiological effects caused by disturbing the relative distribution of Mpc proteins. We therefore constructed strains with a constitutive overexpression of individual Mpc proteins. We first confirmed that the overexpressed Mpc proteins located correctly at the mitochondria (Fig. 4A). We then found that gain of Mpc2 function (and to a lesser degree Mpc1 function) caused inhibition already in minimal glucose growth medium. Both Mpc1 and Mpc2 overexpressing strains were not able to grow on synthetic medium with glycerol as the energy source (Fig. 4B). Furthermore we detected a severe sensitivity to oxidative stress caused by hydrogen peroxide or menadione for both strains (Fig. 4C). Interestingly the Mpc3 overexpressing strain grew normally under non stress conditions and was more tolerant to oxidative stress (Fig. 4C). These data indicated that increased abundance of Mpc2 or Mpc3 antagonistically affected respiratory growth and oxidative stress resistance.

**Figure 5:**
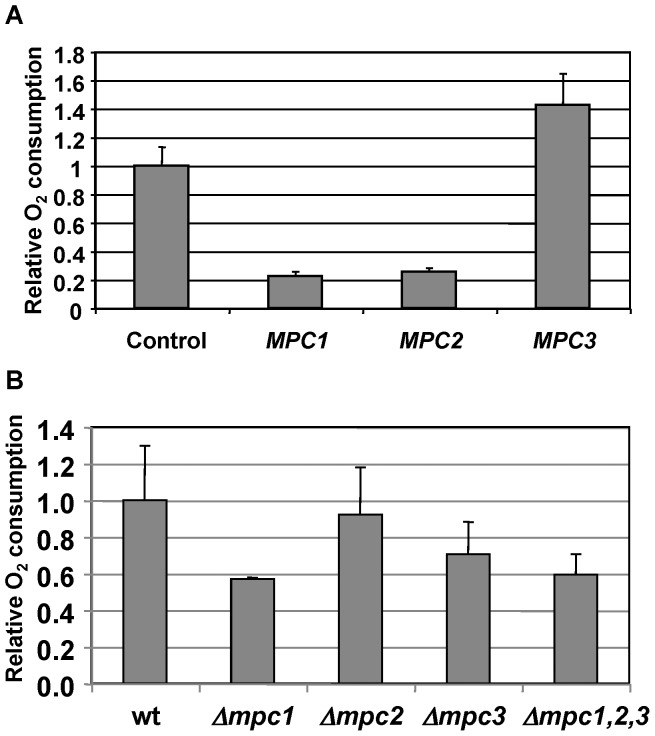
Effects of gain and loss of Mpc function on the oxygen consumption rate. The rate of oxygen consumption was measured in intact yeast cells corresponding to the indicated overexpression (A) or deletion strains (B). The control strain in the upper panel is wild type BY4741 with the empty overexpression vector and untransformed wild type in the lower panel. Data represent mean values +/− SD from at least three independent measurements. Mpc1 and Mpc2 overexpressing strains have significantly reduced oxygen consumption rates (p = 0.02), while Mpc3 overexpressing cells significantly increase the oxygen consumption rate (p = 0.05) according to the Students t-test (A). Oxygen consumption is significantly reduced (p = 0.05) in the *mpc1* and *mpc3* mutants as compared to wt according to the Students t-test (B).

We next tested directly the effect of gain and loss of Mpc function on the respiration rate. Therefore we quantified the oxygen consumption of Mpc overexpression and deletion strains. We found that overexpression of Mpc1 and Mpc2 caused an important decrease in the oxygen consumption rate in intact yeast cells (Fig. 5A). This result correlates well with the slow growth phenotype of both strains under respiratory conditions. Very interestingly, the overexpression of the Mpc3 protein enhanced the respiration rate coinciding with its strong inducibility during diauxic shift and the oxidative stress resistance phenotype observed before. We then quantified the oxygen consumption in the single *mpc* deletion strains and compared it to wild type and the triple *mpc1,2,3* mutant. The respiration rate was diminished in the absence of Mpc1 or Mpc3 function correlating well with the less efficient respiratory growth of both mutant strains (Figures 5B and 3D). The remaining oxygen consumption rate was comparable to the one measured for the full *mpc1,2,3* knockout. No significant reduction of the respiration rate was observed for the *mpc2* mutant (Fig. 5B). Taken together, these results indicated a positive function for Mpc3 in the stimulation of respiration.

**Figure 6:**
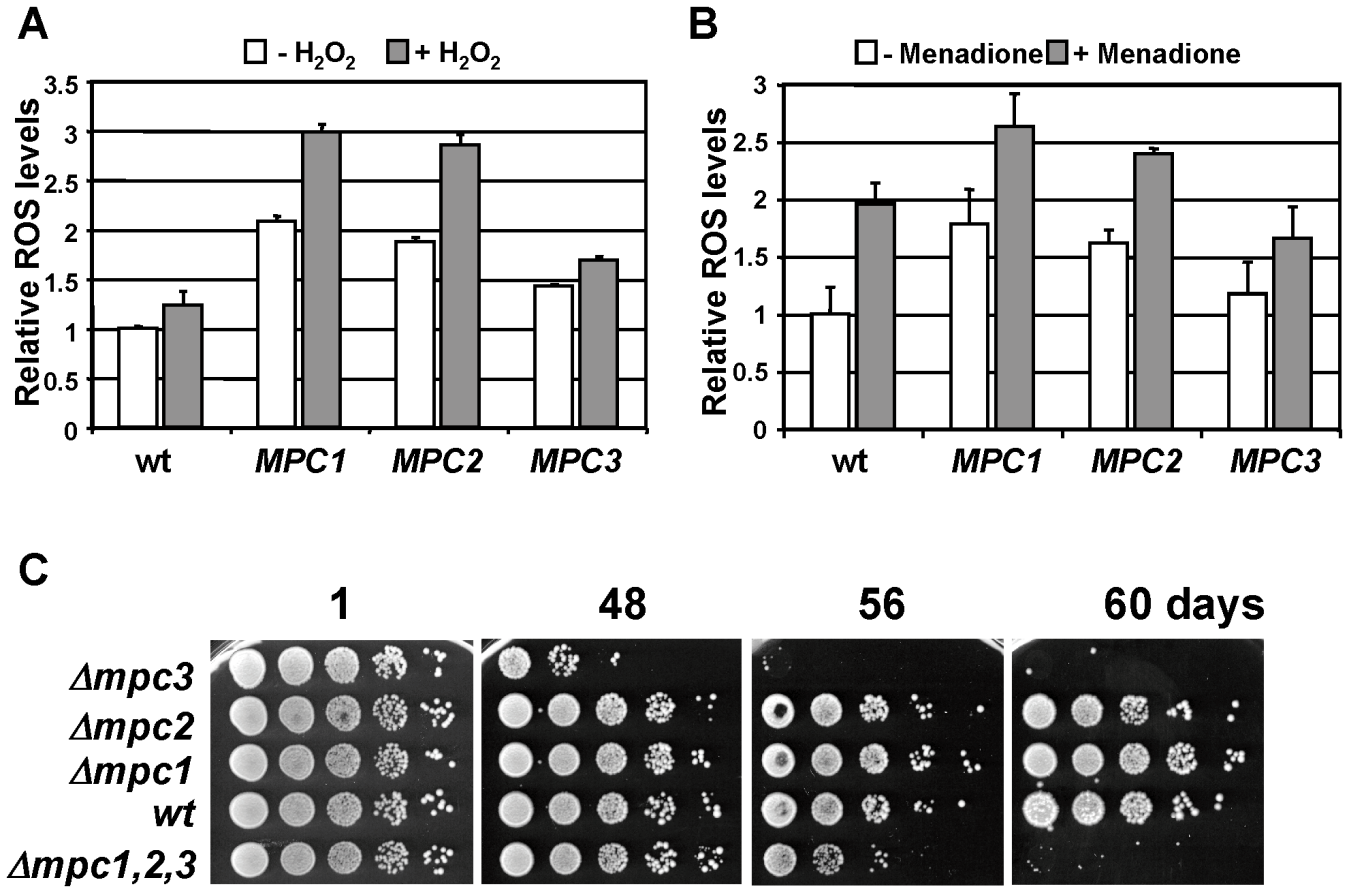
The function of Mpc proteins in ROS balance and survival in stationary phase. A–B. The yeast strains overexpressing individual Mpc proteins described in Fig. 4 were grown in normal SD medium and subjected to a brief oxidative stress caused by hydrogen peroxide (A) or menadione (B). ROS was measured by the oxidation of dichlorodihydrofluorescein as described in “Experimental Procedures”. Fluorescence of the wild type before stress was arbitrarily set to 1. ROS levels were determined for three independent cultures in duplicate. Data presented are mean values +/− SD. Mpc1 and Mpc2 overexpressing strains have significantly increased ROS levels (p = 0.02 for panel A; p = 0.05 for panel B) as compared to wt according to the Students t-test (B). C. The survival in stationary phase in YPD medium of the indicated yeast strains was determined by plate assays after the indicated time.

Having seen that the overexpression of Mpc proteins had different effects on the respiration rate, we next tested whether this had an influence on the ROS balance. We therefore quantified the level of intracellular oxidation under normal and after oxidative stress treatments with hydrogen peroxide and menadione. As shown in Figure 6, Mpc1 and Mpc2 overexpressing strains showed elevated basal and induced ROS levels, while Mpc3 gain of function caused ROS levels comparable to wild type cells. Taken together, Mpc1 and Mpc2 overexpressors are characterized by reduced respiration rates and an overproduction of ROS. We then measured ROS in the individual *mpc* deletion strains. However, we could not detect significantly different ROS levels of either *mpc1*, *mpc2* or *mpc3* mutant strains compared to wild type (Timón-Gómez A., Proft M., Pascual-Ahuir A.; unpublished observation). As the ROS balance is intimately linked to the longevity of yeast cells, we tested the capacity to survive in stationary phase of the *mpc* mutant strains. We found that *mpc3* mutants lost viability during stationary phase much earlier than the wild type, *mpc1* or *mpc2* cells (Fig. 6C). This obvious reduction in the chronological lifespan in the *mpc3* deletion strain was even slightly more pronounced as compared to the triple *mpc1,2,3* mutant.

**Figure 7:**
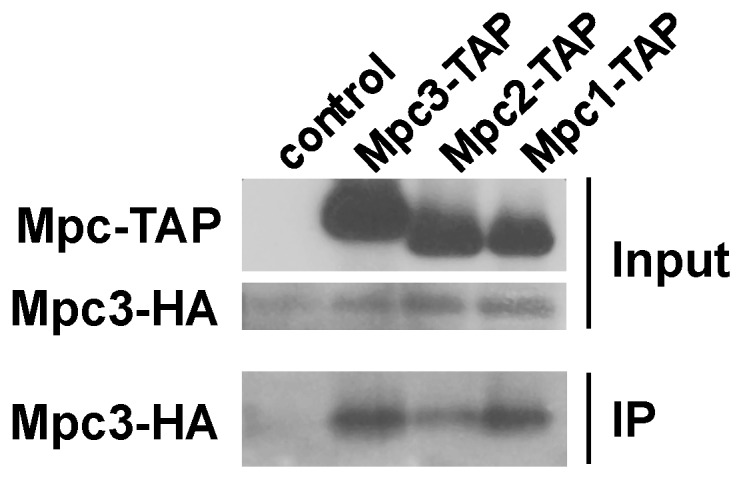
Mpc3 interacts preferentially with Mpc1 and other Mpc3 subunits. Coprecipitation experiments were performed from mitochondrial extracts from yeast cells expressing Mpc3-HA in the presence or not of the indicated Mpc-TAP protein. Immunoblots are shown for the Mpc-TAP and Mpc3-HA proteins in the input and for Mpc3-HA after copurification of the respective TAP-tagged Mpc proteins (IP).

It has been previously concluded from copurification studies that Mpc1 and Mpc2 form a multimeric complex with Mpc2 as the major subunit. Mpc2 is able to both interact with other Mpc2 subunits and with Mpc1 [11]. Here we identify the Mpc3 protein as an inducible component of the mitochondrial pyruvate carrier and we wanted to characterize its interactions with other Mpc proteins. We carried out copurification studies with individually TAP-tagged Mpc proteins in the presence of Mpc3-HA. As depicted in Figure 7, Mpc3-HA preferentially showed interaction with Mpc3-TAP or Mpc1-TAP. A less efficient copurification of Mpc3 with Mpc2 was observed. These data indicated that Mpc3 might preferentially interact with Mpc1 and other Mpc3 subunits.

### Functional Relation between Mpc1, Mpc3 and the Mitochondrial Carnitine Transporter Crc1

**Figure 8:**
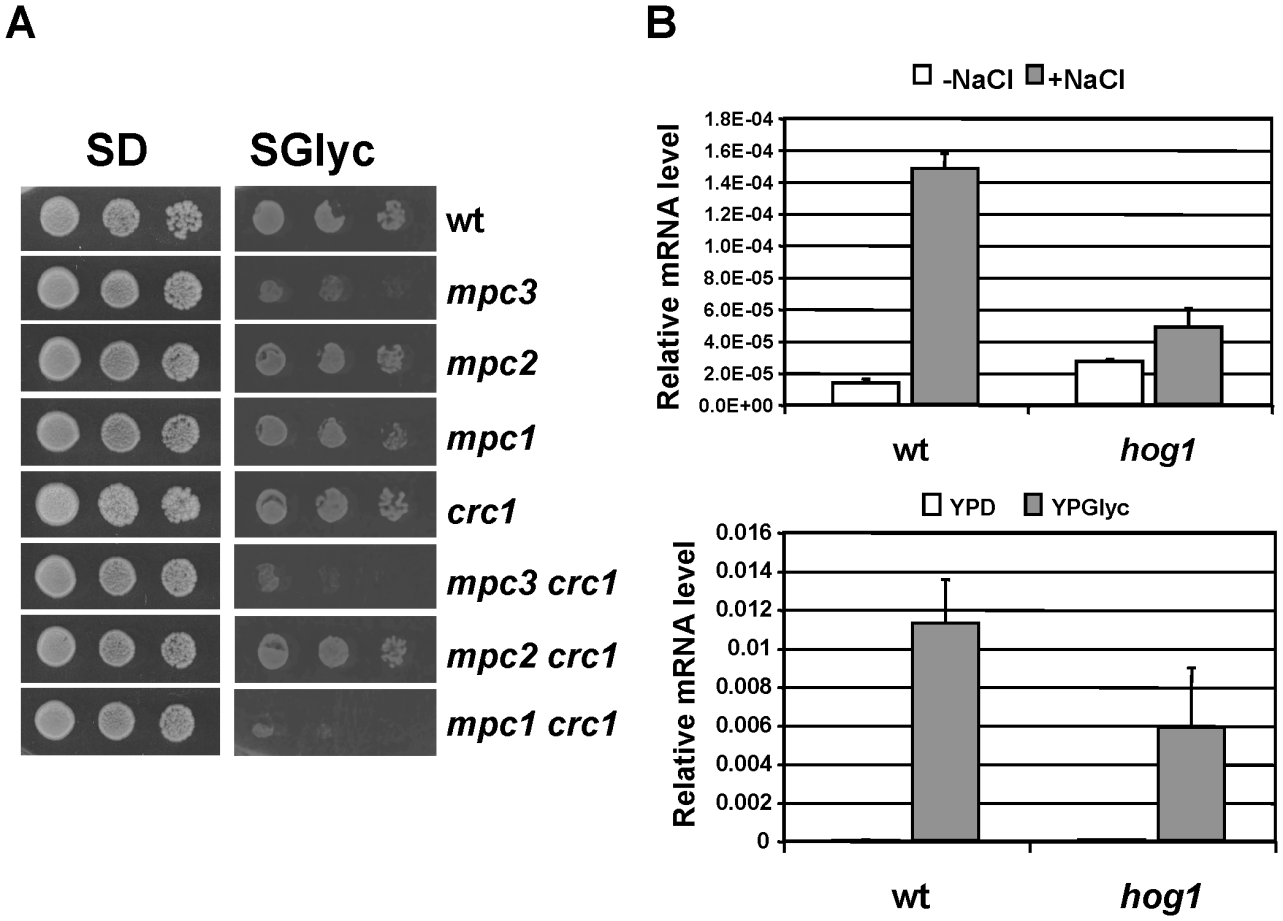
The inducible Crc1 function is important to sustain respiratory growth together with Mpc1 and Mpc3. A. Growth of the indicated yeast strains was assayed on synthetic agar medium containing glucose (SD) or glycerol (SGlyc) as the energy source. Leucine and valine were supplemented to the plates to exclude growth effects caused by diminished mitochondrial amino acid biosynthesis. B. *CRC1* expression is highly activated by osmostress and during the diauxic shift. Yeast wild type (BY4741, wt) and *hog1* mutant strains were treated for 20 min with 0.4 M NaCl (upper panel) or shifted from YPD to YPGlyc medium for 30 min (lower panel). The expression levels were determined by RT-PCR and normalized for the *ACT1* messenger. Data are shown as mean from two independent experiments; error bars represent SD. *CRC1* transcript levels are significantly lower in the *hog1* mutant vs. wild type after NaCl induction (p = 0.003) or diauxic shift (p = 0.05) according to the Students t-test.

We found that respiration rates and growth on a non fermentable carbon source were diminished but not absent in *mpc1* or *mpc3* mutants. This pointed to alternative ways to introduce either pyruvate or acetyl-CoA into mitochondria in the absence of Mpc activity. In yeast, pyruvate import and pyruvate dehydrogenase activity in mitochondria can be bypassed by a carnitine shuttle located at the inner mitochondrial membrane [19], [20]. The yeast mitochondrial carnitine carrier is encoded by the *CRC1* gene and is responsible for the carnitine dependent import of acetyl-CoA, which can be derived by oxidative degradation of pyruvate or fatty acids. We tested whether Crc1 activity contributed to respiratory metabolism together with the Mpc pyruvate carrier. We constructed yeast strains which combined the loss of Crc1 function with deletions of either of the three *MPC* genes. As shown in Figure 8, the decreased growth rate on synthetic non-fermentable medium caused by the lack of Mpc1 or Mpc3 was further diminished by the additional deletion of Crc1. This suggested that Crc1 together with Mpc1,3 was responsible to sustain high respiration capacity. We finally measured whether Crc1 activity was subjected to positive regulation upon osmotic stress or diauxic shift and quantified the *CRC1* expression. We found a very similar expression pattern for *CRC1* as compared to *MPC3* (Fig. 8B). *CRC1* was highly inducible upon osmostress and during the shift to respiratory metabolism, which was dependent on the Hog1 MAP kinase to different degrees. We concluded that the inducible Mpc3 and Crc1 transport functions for pyruvate or acetyl-CoA are necessary for activated respiration rates in yeast cells.

## Discussion

Here we investigate in the yeast model the role of the regulated import of pyruvate into mitochondria in the adjustment of the oxidative metabolism. Our data are in agreement with a model in which the activated expression of the specific subunit Mpc3 of the carrier directs the pyruvate influx towards respiration. In conditions where the oxidation of pyruvate via the tricarboxylic acid cycle (TCA) and the mitochondrial electron transport chain is greatly repressed, the dominant mitochondrial import system for pyruvate is composed of Mpc1 and Mpc2 (Figure 1 and [11], [12]). This is the case for yeast cells in an environment in the presence of fermentable carbohydrates when most of the mitochondrial pyruvate is dedicated to biosynthetic processes such as the synthesis of amino acids. Under these conditions of fermentative growth, the Mpc3 protein is completely dispensable arguing for a function of Mpc3 which is unrelated to amino acid biosynthesis. In turn, Mpc3 protein levels increase upon growth conditions which require the reinforcement of oxidative respiration and it is under these conditions where the loss of Mpc3 causes growth defects (Figure 3). As the end product of glycolysis and once imported into mitochondria, pyruvate is at the crossroad of biosynthetic pathways and oxidative metabolism. Therefore it seems likely that cells have evolved regulatory mechanisms to control the fate of pyruvate according to the demand of new amino acid synthesis and ATP synthesis through oxidative respiration. One such signaling path assures highly regulated expression of the *MPC3* gene. *MPC3* expression is repressed under fermentative non stress conditions and highly induced upon osmostress or by the lack of fermentable carbon sources. Great part of this regulation is due to the activity of the HOG MAP kinase pathway. Accordingly, the *MPC3* gene promoter is directly bound by the Sko1 transcriptional repressor/activator, which is one of the core transcription factors mediating the transcriptional osmostress response downstream of the Hog1 MAP kinase [21], [22]. Since Mpc2 function was specific for amino acid biosynthesis, we additionally investigated whether Mpc2 protein levels were regulated by amino acid starvation. However we were not able to measure any induction of Mpc2 when we compared yeast cells grown on rich glucose medium with amino acid starved conditions (Timón-Gómez A., Proft M., Pascual-Ahuir A.; unpublished observation). Therefore, the tight regulation of Mpc3 along with oxidative metabolism is a specific feature of the Mpc proteins in yeast.

Here we demonstrate in several lines of evidence that disturbing the relative composition of Mpc proteins has important consequences for the respiratory capacity and stress tolerance of the cell. Mutations in single Mpc components indicate that Mpc1 is required for both respiratory growth and efficient amino acid biosynthesis, while loss of Mpc2 or Mpc3 causes selective phenotypes for amino acid biosynthesis or respiration, respectively. In turn, increased abundance of Mpc2 inhibits respiration and raises ROS levels, while Mpc3 causes the opposite effects, an enhanced respiration rate and resistance to oxidative stress. This suggests the existence of specialized pyruvate carrier complexes whose function might be determined by the presence of the Mpc2 or Mpc3 subunits. In line with this hypothesis is the finding that mitochondrial membranes contain a 150 kD complex composed of Mpc1 and Mpc2, which specifically coprecipitate with each other [11], and our finding here that also Mpc3 copurifies preferentially with Mpc1 and Mpc3 subunits. Therefore it will be important in the future to determine whether or not pyruvate import into mitochondria occurs at distinct uptake complexes with different physiological functions.

Yeast cells control the respiration rate in a very tight manner with the help of signaling pathways which in the presence of sugar substrates favor fermentative over oxidative metabolism even when oxygen is available [23]. Here we describe a mechanism which ensures the accumulation of oxidative fuel in form of mitochondrial pyruvate and acetyl-CoA in situations of sugar limitation or stress. This involves the up-regulation of the pyruvate carrier subunit Mpc3 along with alternative transport systems such as the carnitine dependent acetyl-CoA carrier Crc1. Mpc proteins are highly conserved through evolution and might be an interesting therapeutical target to enhance mitochondrial respiration rates in the case of human diseases with deficiencies in the oxidative metabolism of pyruvate [24], [25]. This is especially relevant to understand and manipulate the dependency of tumor cells on fermentative carbon utilization during the Warburg effect [26], [27]. Very interestingly, the human genome contains three *MPC* genes encoding MPC1, MPC2 and MPC1L. MPC1 and MPC1L (orthologs for yeast Mpc1) are >60% identical except a small unrelated C-terminal extension in MPC1L, while MPC2 is the ortholog to yeast Mpc2 and Mpc3. Thus, a regulated and tissue specific distribution of different MPC subunits could also be a mechanism of regulating mitochondrial respiratory capacity in humans.
